# Asymmetric buttocks: think of vascular malformation

**DOI:** 10.11604/pamj.2021.38.81.20986

**Published:** 2021-01-25

**Authors:** Khadija Elboukhari, Mohammed Ouadoud

**Affiliations:** 1 Dermatology Department, Fez University Hospital Center, Fez, Morocco

**Keywords:** Asymmetry, malformation, vascular, fat

## Image in medicine

Vascular malformations are rare vascular diseases leading to severe deformity. They are difficult to diagnose and manage. We here report the case of a 17-year-old female patient with no significant pathological history with congenital patch in the glutes which had been complicated since adolescence leading to a swelling involving the entire homolateral gluteal region, without neurological signs or other signs. Dermatological examination showed gluteal asymmetry and the presence of a large purple angiomatous plaque involving the two-thirds of the internal portion of the right buttock. Clinical examination showed no thrill but a slight increase in local heat on palpation compared with the contralateral side. Flat angioma associated with venous malformation or angiodysplasia was suspected. Doppler ultrasound showed malformation made up of small veins with hypertrophy of the fat tissue. Subsequently, after exlusion of endopelvic involvement, the patient underwent plastic surgery for reduction of fat tissue in the right buttock. In another study that we conducted, involving 120 patients, vascular malformations in the gluteal region were not uncommon. Out of 20 patients 16 had gluteal asymmetry. These malformations were related to age, their prevalence among adolescents (P) between the ages of 11 to 16 was 0,045.

**Figure 1:**
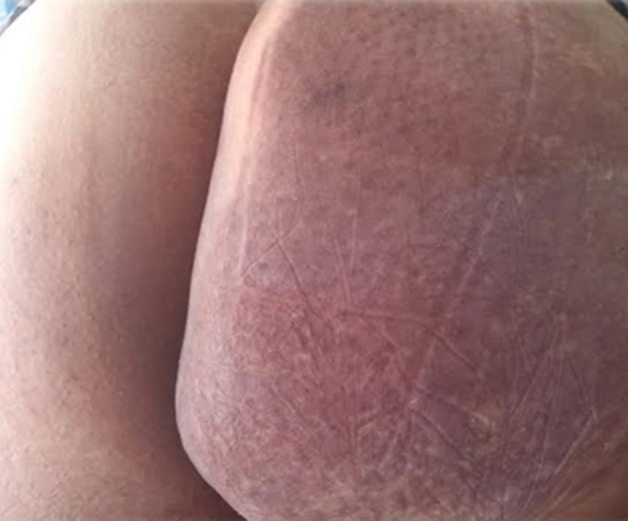
angiomatous patch of the right buttock with an asymmetry of both buttocks

